# Self-Associated 1,8-Naphthalimide as a Selective Fluorescent Chemosensor for Detection of High pH in Aqueous Solutions and Their Hg^2+^ Contamination

**DOI:** 10.3390/s23010399

**Published:** 2022-12-30

**Authors:** Awad I. Said, Desislava Staneva, Silvia Angelova, Ivo Grabchev

**Affiliations:** 1 Faculty of Medicine, Sofia University “St. Kliment Ohridski”, 1407 Sofia, Bulgaria; 2 Department of Chemistry, Faculty of Science, Assiut University, Assiut 71516, Egypt; 3 Department of Textile, Leather and Fuels, University of Chemical Technology and Metallurgy, 1756 Sofia, Bulgaria; 4 Institute of Optical Materials and Technologies “Acad. J. Malinowski”, Bulgarian Academy of Sciences, 1113 Sofia, Bulgaria

**Keywords:** diaminotriazine, self-assembly, 1,8-naphthalimide, fluorescent probe, Hg^2+^, pH, PET, ICT

## Abstract

A novel diamino triazine based 1,8-naphthalimide (NI-DAT) has been designed and synthesized. Its photophysical properties have been investigated in different solvents and its sensory capability evaluated. The fluorescence emission of NI-DAT is significantly impacted by the solvent polarity due to its inherent intramolecular charge transfer character. Moreover, the fluorescence emission quenched at higher pH as a result of photo-induced electron transfer (PET) from triazine moiety to 1,8-naphthalimide after cleaving hydrogen bonds in the self-associated dimers. Furthermore, the new chemosensor exhibited a good selectivity and sensitivity towards Hg^2+^ among all the used various cations and anions in the aqueous solution of ethanol (5:1, *v*/*v*, pH = 7.2, Tampon buffer). NI-DAT emission at 540 nm was quenched remarkably only by Hg^2+^, even in the presence of other cations or anions as interfering analytes. Job’s plot revealed a 2:1 stoichiometric ratio for NI-DAT/Hg^2+^ complex, respectively.

## 1. Introduction

Mercury and its compounds are involved in several important industries such as pharmaceuticals, paints, agricultural chemicals, measuring instruments, chlor-alkali industry, etc. [1]. However, mercury is one of the most toxic heavy metals that threaten the human health and environment. All chemical forms of Hg^2+^ are chronically toxic to humans, especially inorganic derivatives exhibit extremely severe toxicity that leads to a variety of diseases and organs damage [2]. Some organomercurials, particularly low molecular-weight alkyl compounds, are reported to be highly toxic due to their strong affinity to bind with enzymes and proteins leading to mostly irreversible cell dysfunction [3]; nervous system damages [4,5], endocrine disorders [6], as well as failure of the immune [7] and digestive systems [8]. Hence, there is a growing interest in providing new reliable selective and sensitive methods to detect mercury in biological and environmental systems.

Many traditional analytical techniques have recently been applied for detection of mercury ions, namely atomic absorption spectroscopy (AAS) [9], inductively coupled plasma mass spectrometry (ICP-MS) [10], ultraviolet–visible spectrophotometry (UV-vis) [11], surface-enhanced Raman spectroscopy (SERS) [12], surface plasmon resonance (SPR) [13], and electrochemical method [14,15,16]. These techniques ensure the sensitivity, selectivity, and precision of Hg^2+^ detection. However, most of them require sophisticated equipment, complicated and lengthy procedures, and they are also unsuitable for real-time and on-site detections.

Nowadays there has been a growing interest in developing fluorescent compounds for sensory biological or environmental applications because they are easy to be synthesized, highly sensitive and inexpensive [17,18,19,20,21,22,23,24,25,26,27]. Moreover, the ongoing development of confocal microscopy and optical imaging technologies facilitate the utilization of theses fluorescent probes for biological imaging [28,29]. A common mechanism for detecting the presence of Hg^2+^ relies on the fluorescence quenching caused by the spin orbital coupling of Hg^2+^ and the fluorescent molecules [30]. Hence, many of the reported fluorescent probes for the Hg^2+^ determination were designed on the basis of fluorescence emission quenching mechanism [31,32,33].

Different fluorophores in fluorescent chemosensors, such as fluorescein, rhodamine, 1,8-naphthalimides, coumarin, boron-dipyrromethene (BODIPY), pyrene, and anthracene, have been reported in the literature to be suitable for sensors making [34,35,36,37,38]. Most of the scientists’ attention has been attracted by 1,8-aphthalimides as optical sensors due to their light stability, high emission quantum yield, large Stokes shifts, and considerable dependence of their absorption and emission on the surrounding environment [39,40,41,42,43,44,45,46,47,48]. Some fluorescent chemosensors for detecting Hg^2+^ have recently been reported. However, most of them have the disadvantage of being insoluble in aqueous media, of low sensitivity, selectivity, and obtained by complicated synthetic procedures [49,50,51,52,53,54,55,56,57,58,59,60,61].

2,4-Diamino-1,3,5-triazines (DATs) are well-known to have a potential chemotherapeutic activity especially for treating malignant neoplasms-leukemia, melanoma, and breast cancer [62,63,64]. Furthermore, due to the high nitrogen content they have a good affinity for complexing with metals [65]. Besides, diaminotriazines are well-known to have a donor–acceptor–donor (DAD) feature facilitating self-associations in solutions, presumably by forming hydrogen-bonded dimers or higher-order aggregates [66,67,68]. 

Herein, we report on the synthesis of a new 1,8-naphthalimide modified with 4,6-diamino-1,3,5-triazine and on its capacity to detect various cations and anions based on its photophysical properties in various solvents of different polarity. The influence of pH in H_2_O:ethanol (5:1, *v*/*v*, pH = 7.2, Tampon buffer) solutions has been investigated as well.

## 2. Materials and Methods

All used fine chemicals and solvents were of spectroscopic grade purity. ^1^H spectrum was recorded on a Bruker Avance II + 600 spectrometer (Bruker BioSpin GmbH, Rheinstetten, Germany) operating at 600.13 MHz using DMSO-d_6_ as a solvent at 25 °C. Absorption and emission spectra were recorded on a Varian Cary 5000 UV-Vis-NIR Spectrophotometer and a “Cary Eclipse” fluorometer (Agilent Technologies Deutschland GmbH, Darmstadt, Germany), respectively, using quartz cuvettes (Hellma, Munich, Germany). Origin pro 8 software was used for processing the absorption and emission data. The reaction was monitored on a TLC (Fluka F_60_ 254 20 × 20; 0.2 mm) with a 4:1 toluene:methanol solution as an eluent. TLC plates were investigated under UV light. Melting points were measured using a Hinotek-X4 micro melting point apparatus (Hinotek, Ningbo, China). All 2D structures were drawn using Chem3D ultra 9.0 (Chem Office 2008) program. A detailed description of the adopted computational protocol for the DFT calculations is given as Supplementary Information.

### 2.1. Synthesis of 4-Nitroacenaphthene (***2***)

A solution of acenapthene (10 g, 66 mmol) in dichloromethane (50 mL) was added to a 250 mL two-necked flask and cooled to 5 °C. Then, 12 mL nitric acid (48%) were added dropwise over 30 min. The mixture was stirred vigorously for 4 h. The resulting precipitate was filtered and dried. Yield: 95%, mp 101–103 °C (lit. 101 °C) [69,70]. 

### 2.2. Synthesis of 4-Nitro-1,8-naphthalic anhydride (***4***)

4-Nitroacenaphthene (10 g, 50 mmol) were added to glacial acetic acid (100 mL) in a three-necked flask and heated to 95 °C. Then, sodium bichromate (58 g, 0.2 mol) were added in portions. The reaction temperature was kept 95 °C through the addition. After stirring for 4 h at 95 °C, the reaction mixture was poured into cold water (0.5 L) and the resulting precipitate was filtered off, washed with water, and dried. The crude product was treated with 150 mL aqueous NaOH (5%), filtration, and then the pH of the filtrate was adjusted to 4 using conc. HCl. The resulting precipitate was filtered, washed with water, and dried to give 3, which was dehydrated by heating at 130 °C for 10 h to give 4. Yield: 70%, mp 230–233 °C (lit. 233 °C) [70].

### 2.3. Synthesis of 4-Amino-1,8-naphthalic anhydride (***5***)

To an emulsion of 4 (1 g, 4 mmol) in ethanol (25 mL), a solution of stannous chloride (6.2 mg, 24 mmol) in 8 mL concentrated hydrochloric acid (8 mL) was added dropwisely at room temperature. Then, the reaction was refluxed for 10 h. After cooling, the hydrochloric acid was neutralized using an aqueous solution of Na_2_CO_3_ (10%). The formed precipitate was collected by filtration, washed with water, and dried. Yield: 60%, mp 352–357 °C (lit. 358 °C) [71].

### 2.4. Synthesis of 4-Amino-N-(2-dimethylaminoethyl)-1,8-naphthalimide (***6***)

To a solution of 4-amino-1,8-naphthalic anhydride (0.5 g, 2.3 mmol) in ethanol (25 mL), *N*,*N*-dimethylethylenediamine (0.5 mL, 4.2 mmol) was added dropwise with stirring, then refluxed for 6 h. The solvent was removed under reduced pressure to yield the final product. Yield: 98%, mp 226–230 (lit. 223–225) [72].

### 2.5. Synthesis of 4-(4,6-Diamino-1,3,5-triazin-2′-ylamino)-N-(2-dimethylaminoethyl)-1,8-naphthalimide NI-DAT

4-Amino-*N*-(2-dimethylaminoethyl)-1,8-naphthalimide (0.28 g, 1 mmole) and 2-chloro-4,6-diamino-1,3,5-triazine (0.15 g, 1 mmole) were refluxed in ethanol (25 mL) till reaction completion (6 h) which was confirmed by TLC. The pure product was obtained after filtration and washing with ethanol. Yield 85 %, 0.33 g, mp 235–239 °C. 

FT-IR (KBr) cm^−1^: 3360, 3220 (νNH_2_) 3100 (νCH (Aromatic)); 2922, 2857 (νCH (Aliphatic)); 1698, 1622 (νC = O). ^1^H NMR (600 MHz, DMSO) δ 10.30 (d, *J* = 9.4 Hz, 2H (dimer NH_2_), 9.78 (d, *J* = 9.2 Hz, 1H, dimer ArH), 9.33 (d, *J* = 9.7 Hz, 2H, dimer NH_2_), 9.15 (d, *J* = 9.8 Hz, 1H, dimer ArH), 8.62–8.57 (m, 1H, monomer ArH), 8.55 (d, *J* = 8.2 Hz, 1H, dimer ArH), 8.42 (d, *J* = 6.5 Hz, 1H, monomer ArH), 8.34 (d, *J* = 6.2 Hz, 1H, dimer ArH), 8.17 (d, *J* = 6.6 Hz, 1H, monomer ArH), 8.10 (d, *J* = 7.6 Hz, 1H, dimer ArH), 7.67–7.60 (m, 1H, monomer ArH), 7.60–7.54 (m, 1H, monomer ArH), 7.43 (s, 1H, monomer > NH), 7.38 (s, 2H, dimer > NH), 6.86–6.74 (m, 8H, 4 dimer NH_2_ & 4 monomer NH_2_), 4.23 (d, *J* = 27.7 Hz, 6H, 4 dimer CH_2_ & 2 monomer CH_2_), 3.95–3.74 (m, 6H, 4 dimer CH_2_ & 2 monomer CH_2_), 3.05 (s, 6H, 2 monomer CH_3_), 3.03 (s, 12H, 4 dimer CH_3_).

## 3. Results and Discussion

### 3.1. Design and Synthesis of NI-DAT

**Scheme 1:**
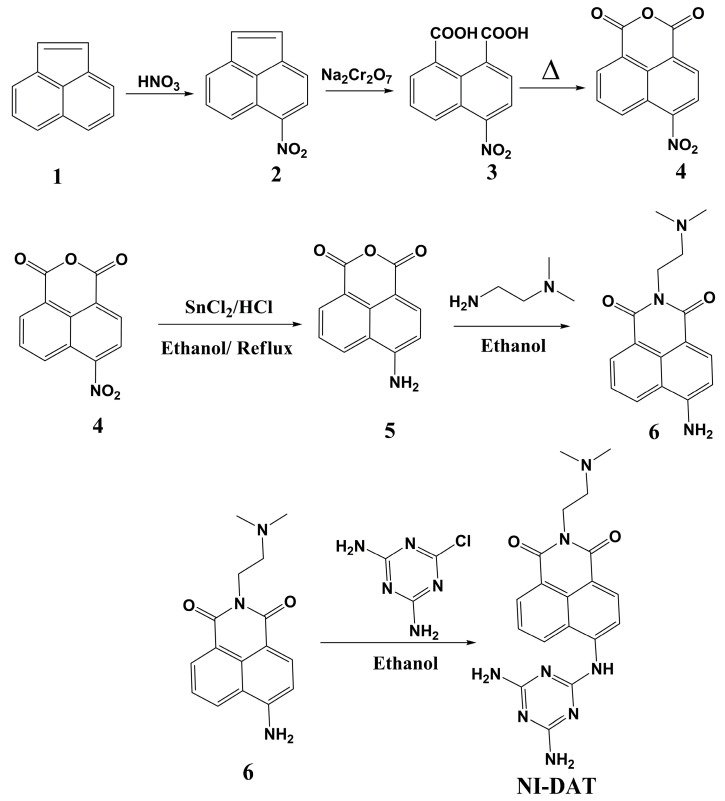
Synthesis of NI-DAT.

**Figure 1:**
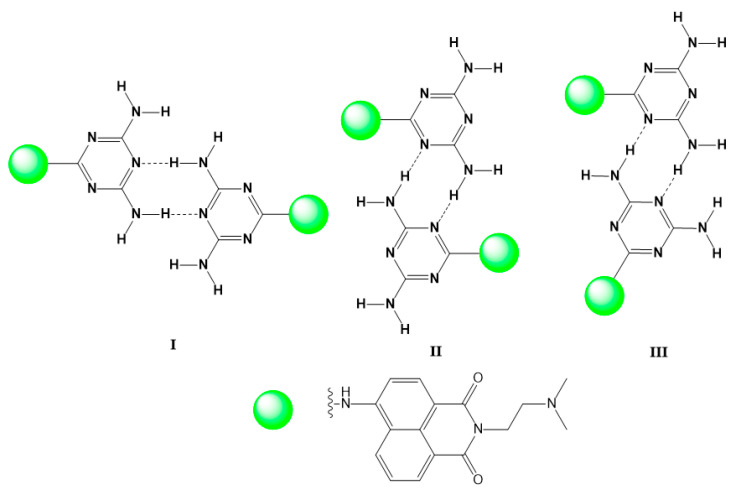
Hydrogen bonding motifs formed by self-assembling dimers of DAT residues.

4-(4,6-diamino-1,3,5-triazin-2′-ylamino)-N-(2-dimethylaminoethyl)-1,8-naphthal-imide NI-DAT was prepared according to Scheme 1, presenting the nucleophilic substitution of 2-chloro-4,6-diaminotriazine by a 4-amino-*N*-(2-dimethylaminoethyl)-1,8-naphthalimide. The chemical structure of NI-DAT was confirmed by IR, NMR absorption, and emission spectra. ^1^H-NMR spectrum, (see the Supplementary Information), confirms the formation of a stable self-associated dimer of the compound. The design of NI-DAT is based on appending 4,6-diaminotriazine, a well-known chelating moiety, to 4-amino-1,8-naphthalimide, whose photophysical properties are influenced by various stimuli. Moreover, the donor-acceptor-donor feature of DAT that leads to self-associations in solutions (presumably by forming hydrogen-bonded dimers I–III, Figure 1), blocks PET triggering in the system that can be retrieved by stimuli able to separate the probe molecules of their dimers. 

### 3.2. Photophysical Characteristics

Table 1 presents the photophysical characteristics of NI-DAT in solvents of different polarity: absorption (*λ_A_*) and emission maxima (*λ_F_*), Stokes shifts (*ν_A_* − *ν_F_*), and quantum yield of florescence (Φ*_F_*) using fluorescein as a standard (Φ*_st_* = 0.79 in ethanol). The fluorescence quantum yield was calculated on the basis of the absorption and fluorescence spectra of NI-DAT by Equation (1).

(1) $$\Phi_{F}=\Phi_{st}\frac{S_{u}}{S_{st}}\frac{A_{st}}{A_{u}}\frac{n_{u}{}^{2}}{n_{st}{}^{2}}$$

where Φ*_F_* is the emission quantum yield of the sample; Φ*_st_* is the emission quantum yield of fluorescein as standard (Φ*_st_* = 0.79 in ethanol), *A_st_* and *A_u_* represent the absorbance of the standard and sample at the excitation wavelength (*λ_ex_*_._ = 450 nm), respectively; while *S_st_* and *S_u_* are the integrated emission band areas of the standard and sample, respectively, and *n_st_* and *n_u_* are the solvent refractive index of the standard and sample; subscripts *u* and *s* refer to the unknown (sample) and standard, respectively. Stokes shifts were calculated using Equation (2).

(2) $$vA-vF=\left(\frac{1}{\lambda_{A}}-\frac{1}{\lambda_{F}}\right)\times 10^{7}$$

**Figure 2:**
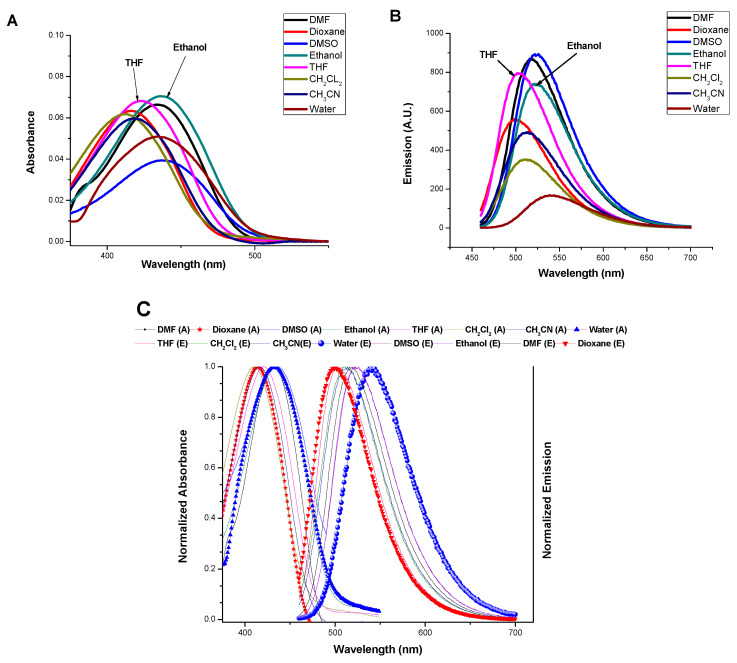
(**A**) Absorption spectra, (**B**) emission spectra, and (**C**) Normalized absorption and emission spectra of NI-DAT in various solvents. *λ_ex_*_._ = 450 nm.

**Figure 3:**
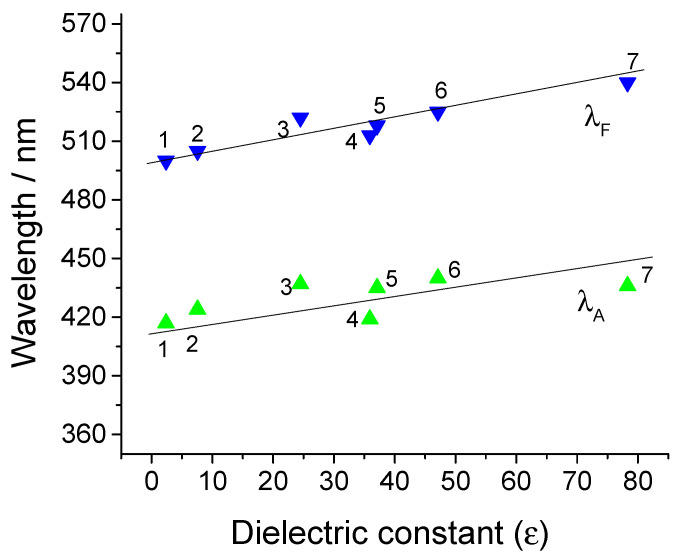
Dependence of absorption (green triangles) and fluorescence (blue triangles) maxima of NI-DAT on the dielectric constant: 1—Dioxane; 2—Tetrahydrofuran; 3—Ethanol; 4—Acetonitrile; 5—*N*,*N*-dimethylformamide; 6—Dimethyl sulfoxide; 7—Water.

As shown in Figure 2, the new NI-DAT has an emission band centered at 500–540 nm which is a mirror image of the corresponding absorption band centered at 417–440 nm. The observed bathochromic shift of the absorption and emission maxima upon increasing the solvent polarity (Figure 2 and Figure 3) was ascribed to the charge transfer as a result of dipole-dipole interactions of the solvents with 1,8-naphthalimide chromophore system. That fact is in good accordance with the characteristics of the reported 1,8-naphthalimide containing compounds [73,74,75,76]. The red-shifts of the emission were higher than their absorption counterparts due to the large difference in the dipole moments in the excited and ground states. 

### 3.3. Impact of the Medium pH

**Figure 4:**
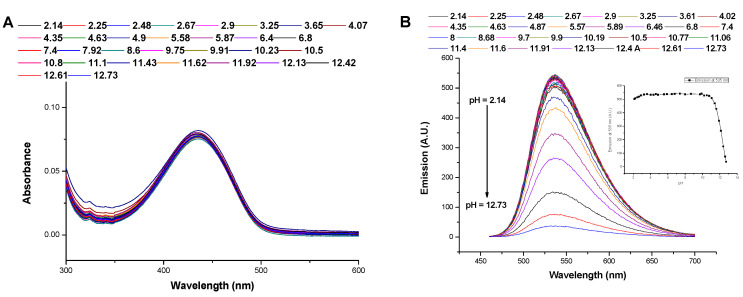
Influence of pH on (**A**) the absorption spectrum (**B**) the emission spectrum and on the emission at 535 nm (inset) of NI-DAT, (c = 10^−5^ M), *λ_ex_*_._ = 450 nm.

To investigate the effect of pH of the medium on the photophysical properties, the absorption and emission spectra of NI-DAT have been measured at different pH of the water/ethanol (5:1, *v*/*v*) solution. As shown in Figure 4, the absorption spectrum didn’t change by varying the pH. On the other hand, the fluorescence emission quenched at higher pH due to the deprotonation of NH_2_ at pKa = 12.1 which was calculated using the Henderson–Hasselbach Equation (3).

(3) $$pH=pK_{a}+\log\frac{\left(I_{max}-I\right)}{\left(I-I_{min}\right)}\;$$

where *I_max_* and *I_min_* are maximum and minimum fluorescence intensity, respectively; *I* is the fluorescence intensity at the given pH value.

**Scheme 2:**
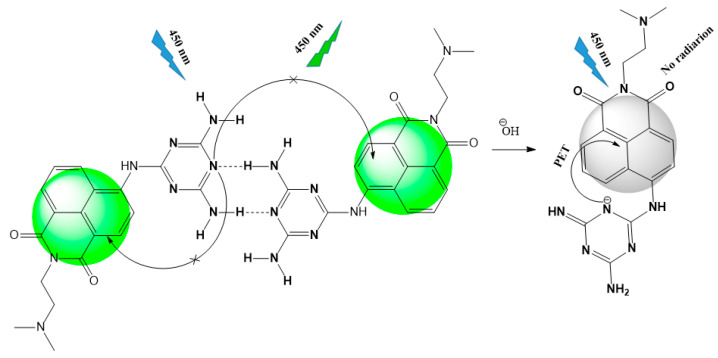
Proposed PET mechanism at different pH.

At lower pH, the inclusion of nitrogen atoms (imine nitrogen of triazine moiety) in the hydrogen bonding of the dimer assembly, shown in Figure 1, blocks PET to 1,8-naphthalimide and hence opens fluorescence emission like the related reported 1,8-naphthalimides analogs [77,78,79,80,81]. In contrast, the probe responds to higher pH by an on-off response due to the deprotonation of NH_2_ and concomitant switching of PET, Scheme 2. 

As Figure 4B shows, PET blocking of the probe at neutral pH can be exploited for further sensory applications of cations and/or anions detection. Coordination with a specific cation may lead to breaking the dimer assemblies giving a selective on-off PET response induced by the recovery of PET to naphthalimide moiety. 

### 3.4. Sensory Applications towards Cations and Anions

**Figure 5:**
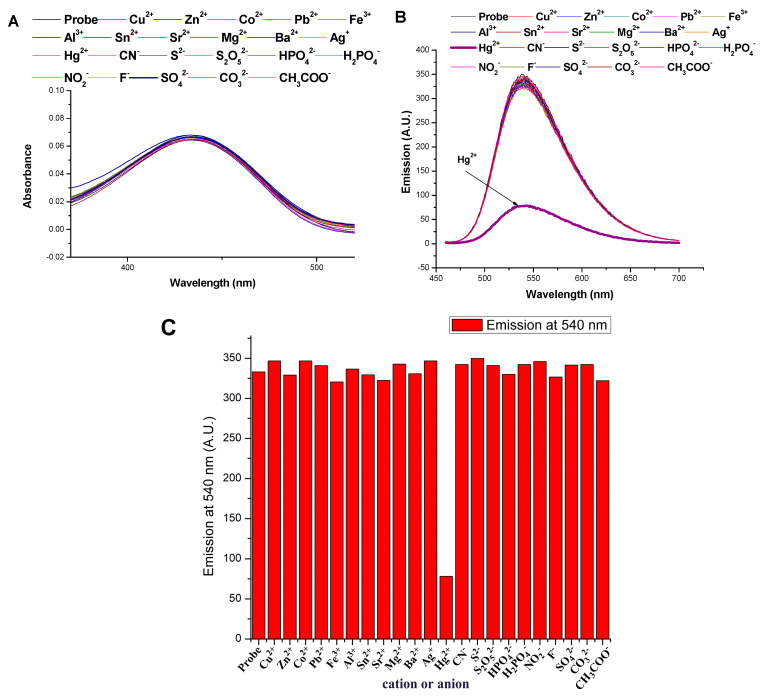
Influence of cations and anions on (**A**) the absorption spectrum and (**B**) the emission spectrum, and (**C**) the emission at 540 nm of NI-DAT, c = 10^−5^ M, *λ_ex_*. = 450 nm.

The sensor ability of NI-DAT towards cations and anions has been investigated by recording the absorption and emission spectra in the presence of cations or anions. Several cations, including Cu^2+^, Co^2+^, Hg^2+^, Zn^2+^, Ni^2+^, Pb^2+^, Sn^2+^, Sr^2+^, Ba^2+^, Mg^2+^, Fe^3+^, and Al^3+^ (as nitrate salts), and different anions, including CN^−^, S^2−^, HPO_4_^2−^, H_2_PO^4−^, F^−^, S_2_O_5_^2−^, SO_4_^2−^, NO_2_^−^, CO_3_^2−^, and CH_3_COO^−^ (as sodium salts), have been used in the study. Moreover, the experiments have been performed using aqueous solution of NI-DAT (5:1 water:ethanol) in the presence of a Tampon buffer to keep a constant pH (7.4) and the variations in the spectra due only to the coordination with the added analyte. As shown in Figure 5, among all the used cations, only Hg^2+^ affected the emission of the probe where it quenched its emission (EQ = 77%). This emission quenching (EQ) has been ascribed to the coordination of Hg^2+^ with NH_2_ groups switching the PET to 1,8-naphthalimide from triazine nitrogens. On the other hand, none of the cations under study significantly affected the absorption spectrum, which confirms that NH- group, attached directly to 1,8-naphthalimide, does not participate in the coordination with Hg^2+^.

**Figure 6:**
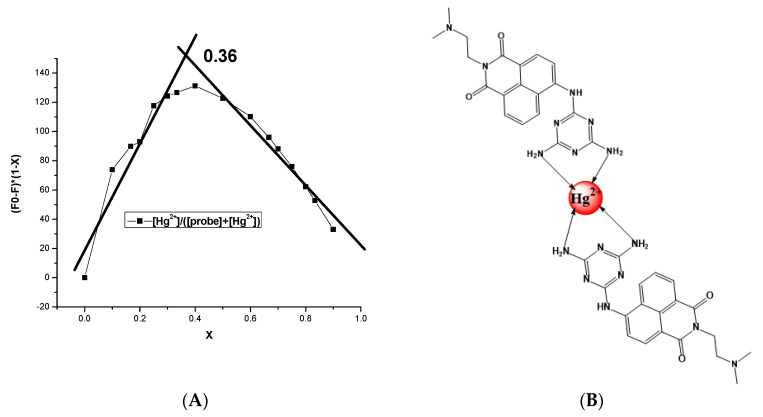
(**A**) Job’s plot and (**B**) suggested structure of probe/Hg^2+^ complex.

The stoichiometric ratio of Hg^2+^ and the probe in their complex was determined using Job’s plot analysis by plotting (F_0_ − F) (1 − X), where F and F_0_ are the emissions of the probe in the absence and presence of Hg^2+^, respectively, against the molar ratio of Hg^2+^ (X = [Hg^2+^]/([NI-DAT] + [Hg^2+^]). As shown in Figure 6, the stoichiometric ratio was found to be 2:1 for the complexion of NI-DAT and Hg^2+^, respectively.

**Figure 7:**
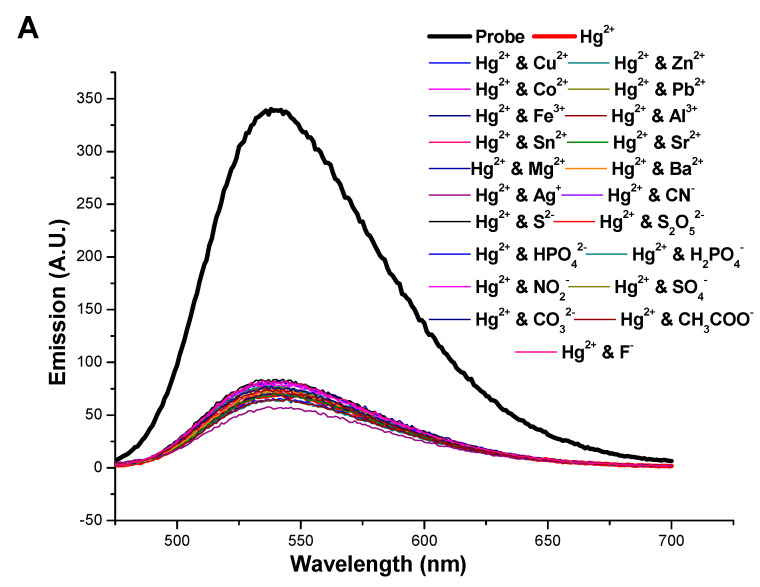

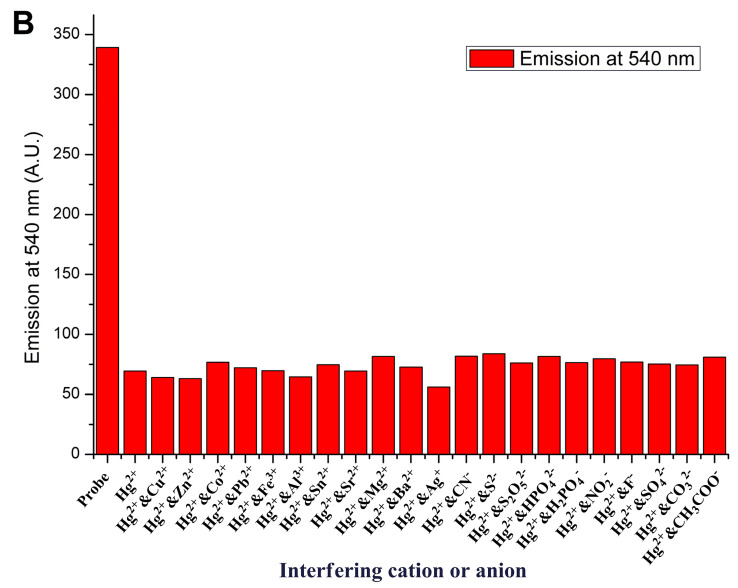
Influence of the interfering cations and anions on (**A**) the emission spectrum and (**B**) emission at 540 nm of NI-DAT, c = 10^−5^ M, *λ_ex._* = 450 nm.

Furthermore, the selectivity of the new chemosensor to Hg^2+^ response has been examined by measuring the emission spectrum of the probe in the presence of 5 equivalents of both Hg^2+^ and the interfering cation or anion. As shown in Figure 7, the emission response of the probe toward the presence of Hg^2+^ was not affected by the coexistence of any of the interfering cations or anions under the study.

**Figure 8:**
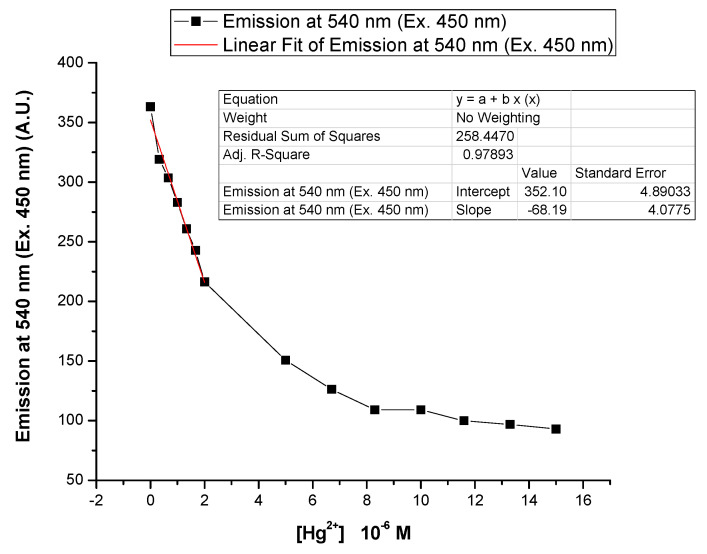
Effect of Hg^2+^ concentration on the emission at 540 nm of NI-DAT, c = 10^−5^ M, *λ_ex_*_._ = 450 nm.

The sensitivity of NI-DAT to detect Hg^2+^ has been estimated from the titration plot of the emission at 540 nm against the concentration of Hg^2+^, shown in Figure 8, using the formula of the limit of detection (LOD) = 3*σ*/*b*, where *σ* is the slandered deviation of the emission of the probe recorded at 540 after repeating the measurements 10 times, and *b* is the slop of the titration plot. The found LOD was found to be 2 × 10^−7^ M. 

### 3.5. Computational Studies on the Structure of NI-DAT and Its Hg^2+^ and Mg^2+^ Complexes

The titled compound NI-DAT was investigated theoretically by means of DFT calculations. A full geometry optimization of the sensor molecule was performed at B3LYP/6-31+G(d,p) level of theory in the gas phase (ε = 1.0). The gas phase optimized structure of the low-energy isomer of NI-DAT is visualized in Figure S3A. The coordination mode between the sensor molecule and Hg^2+^ ions was probed computationally by modeling a ligand/metal architecture from simplified ligand model–2,4,6-triamino-1,3,5-triazine (TAT). TAT was also used in modeling H-bonded dimeric structures, and the intermolecular NH…N distances were measured to be 2.02 Å (Figure S3B). 

**Figure 9:**
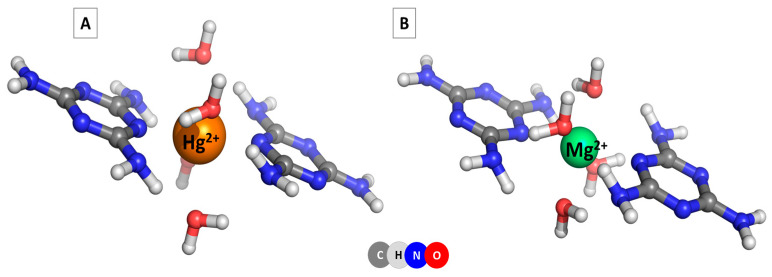
TAT-hydrated metal complexes at 2:1 stoichiometry: (**A**) TAT-hydrated Hg^2+^ complex and (**B**) TAT-hydrated Mg^2+^ complex.

The stoichiometry proposed for the binding between NI-DAT and Hg^2+^ is 2:1 (Figure 5, Job’s plot experiment). Our complex was modeled by placing Hg^2+^ spatially close to one of the endocyclic N atoms of TAT for a complex with 2:1 (TAT:metal) stoichiometry. It is known that Hg^2+^ is hydrated by a first solvation shell of six water molecules [82]. Thus, the TAT:metal complex was modeled with a six coordinate octahedral geometry with four water molecules at the metal cation. Figure 9A depicts the simplified model TAT complexing Hg^2+^ as a monodentate ligand at 2:1 stoichiometry.

In the 2:1 complexes, each model ligand is coordinated to the metal cation via a single nitrogen bridging atom. The metal cation has a six-coordinate octahedral geometry. Each water molecule is bound to two amino groups (one from each of the two ligands). 

The Gibbs energies of the complex formation reaction 2TAT + [Hg(H_2_O)_6_]^2+^ → TAT@[Hg(H_2_O)_4_]^2+^@TAT + 2H_2_O are given in Table 2. The results for the Gibbs energy of the complex formation reaction with Hg^2+^ indicate spontaneous and energy-favorable complex formation processes in the gas phase and in water environment.

In addition, possible complexes formed as a result of the complexation of hexahydrated Mg^2+^ cations, [Mg(H_2_O)_6_]^2+^, with TAT model system were modeled. The positive value (11.9 kcal mol^−1^) for the Gibbs energy of the complex formation reaction with hydrated magnesium ions in water indicates non-spontaneous and energy-unfavorable complex formation processes. Magnesium ion coordinates six water molecules more strongly than mixed ligand (TAT)/water molecules. The results obtained for the complexation of hydrated Hg^2+^ and Mg^2+^ cations with a simplified NI-DAT model are consistent with the experimental data and outline a difference in the complexation behavior of metal cations from the ligand.

### 3.6. Comparison between NI-DAT and Some of the Reported Sensors for Detecting Hg^2+^

A comparison between the NI-DAT chemosensor and some of the reported sensors for detecting Hg^2+^ is shown in Table 3. NI-DAT can be considered as a good candidate for detecting Hg^2+^ due to its good solubility in water and low limit of detection.

## 4. Conclusions

The present work reporting on a simple appending of 2,4-diaminotriazine to 4-amino-1,8-naphthalimide has demonstrated that NI-DAT is able to form in aqueous solution stable self-associated dimers by hydrogen bonding. This hydrogen bonding enhances the emission of the probe by blocking PET from triazine moiety to 1,8-naphthalimide. At higher pH, the hydrogen bonding does not occur due to NH_2_ deprotonation. Hence, the PET is retrieved, and the emission quenched. Moreover, Hg^2+^ selectively and sensitively quenches the fluorescence emission of the probe solution due to its binding to the probe through NH_2_ groups that detach the probe molecules from their dimers. The stoichiometric ratio of NI-DAT/Hg^2+^ complex was found to be 2:1, respectively. The coordination behavior of the studied sensor molecule towards metal ions is supplemented with computational (DFT) data and possible structures of Hg^2+^ and Mg^2+^ complexes in water solutions are proposed. 

## Figures and Tables

**Table 1:** Photophysical characterization of NI-DAT in various organic solvents.

	Dielectric Constant (25 °C)	*λ_A_* nm	*ε* (mol^−1^ L cm^−1^)	*λ_F_* nm	*ν_A_* − *ν_F_* cm^−1^	Φ*_F_*
Water	78.35	436	5224	540	4417	0.359
DMSO	47.1	440	4993	525	3680	0.783
*N*,*N*-dimethylformamide	37.5	435	5879	518	3683	0.828
Acetonitrile	36.71	419	5869	513	4373	0.717
Ethanol	24.5	437	7304	522	3726	0.604
Tetrahydrofuran	7.58	424	6918	505	3783	0.874
Dioxane	2.25	417	5845	500	3981	0.542

**Table 2:** Gibbs energies for the complex formation reactions with hydrated Hg^2+^ and Mg^2+^ cations in the gas phase (ΔG^1^) and in water environment (ΔG^78^), in kcal mol^−1^.

Reaction	ΔG^1^	ΔG^78^
2TAT + [Hg(H_2_O)_6_]^2+^ → TAT@ [Hg(H_2_O)_4_]^2+^@TAT + 2H_2_O	−44.1	−13.6
2TAT + [Mg(H_2_O)_6_]^2+^ → TAT@ [Mg(H_2_O)_4_]^2+^@TAT + 2H_2_O	−22.9	11.9

**Table 3:** Comparsion of some fluorescent chemosensors for Hg^2+^ detection.

Ref.	Sensor	Solvent	LOD 10^−6^ M
[50]	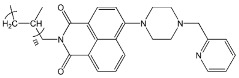	water	2.0
[51]	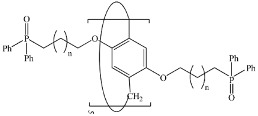	C_2_H_5_OH/H_2_O (3/2)	2.4
[52]	DNA-templated alloy Ag-Au nanoparticles	-	5
[53]	Cationic polydiacetylene	-	8.3
[54]	Gold Nanoparticles		1
[55]	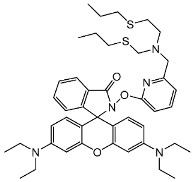	Ethanol/water (4:1)	2.4
[56]	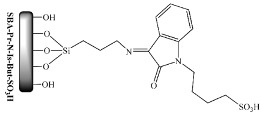	EtOH/H_2_O (5:95)	2.5
[57]	porous triazene-based Cd-MOF	DMF Unstable in water	0.22
[58]	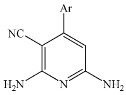	Ethanol	149
[59]	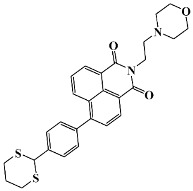	ACN: H_2_O (8:2)	0.003
[60]	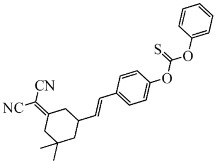	EtOH	6
[61]	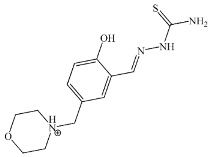	water	0.5
Current work	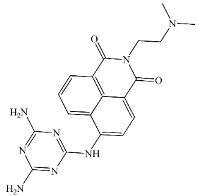	water/ethanol 5:1	0.2

## Data Availability

The data presented in this study are available on request from the corresponding author.

## Supplementary Materials

The NMR and IR spectra of NI-DAT can be downloaded at: https://www.mdpi.com/article/10.3390/s23010399/s1, Figure S1. ^1^H-NMR of NI-DAT; Figure S2. FTIR spectrum of NI-DAT; Figure S3. B3LYP/6-31+G(d,p) optimized structure of (A) NI-DAT and (B) dimer of TAT. References [83,84,85,86,87,88,89] are cited in the Supplementary Materials.
