# Supplementary material for: Self-Associated 1,8-Naphthalimide as a Selective Fluorescent Chemosensor for Detection of High pH in Aqueous Solutions and Their Hg2+ Contamination
Source: Sensors (Basel). 2022 Dec 30;23(1):399. doi: 10.3390/s23010399 (PMC9824833; doi:10.3390/s23010399)
Supplement: Supplementary file 1 [file sensors-23-00399-s001.zip › sensors-2082939-supplementary.pdf]

Supporting Information

Self-Associated 1,8-Naphthalimide as a Selective Fluorescent Chemosensor for Detection of High pH in Aqueous Solutions and Their Hg^{2+} Contamination

Awad I. Said [1,2,*], Desislava Staneva [3], Silvia Angelova [4] and Ivo Grabchev [1,*]

1 Faculty of Medicine, Sofia University "St. Kliment Ohridski", 1407 Sofia, Bulgaria
2 Department of Chemistry, Faculty of Science, Assiut University, Assiut 71516, Egypt
3 Department of Textile, Leather and Fuels, University of Chemical Technology and Metallurgy, 1756 Sofia, Bulgaria; grabcheva@mail.bg
4 Institute of Optical Materials and Technologies "Acad. J. Malinowski", Bulgarian Academy of Sciences, 1113 Sofia, Bulgaria; sea@iomt.bas.bg
* Correspondence: awadsaid@aun.edu.eg (A.I.S.); i.grabchev@chem.uni-sofia.bg (I.G.)

All DFT calculations were performed using Gaussian 09 (G09) computational chemistry software package [1]. The molecular geometries of NI-DAT and TAT were fully optimized at B3LYP/6-31+G(d,p) level using B3LYP [2,3] functional and a diffuse function-augmented version of 6-31G(d,p) [4,5] Pople's basis set for the lighter atoms and SDD pseudopotential for Hg. Default G09 optimization algorithm and convergence criteria were applied. C1 symmetry (i.e., no symmetry) was assumed for all systems. Local minima were verified by establishing that the Hessians had only positive eigenvalues. Single point calculations were performed in water using the IEFPCM (Integral Equation Formalism Polarizable Continuum Model [6]) method.

The differences ΔE_{el}, ΔE_{th}, $P\Delta V$ (work term), and ΔS between the products of the complex formation reactions (complexes) and reactants (TAT ligands and metal cations) were used to evaluate the Gibbs energy of the complex formation in water, ΔG^{78} (ε^{water}=78), at T= 298.15 K by the thermochemical values calculated in Gaussian based on the harmonic vibrational frequencies according to the equation:

$$\Delta G^{78} = \Delta E_{el} + \Delta E_{th} + P\Delta V - T\Delta S \quad (1)$$

where E_{el} is electronic energy, E_{th} thermal energy, P pressure (1.0 atm), V volume, and S entropy.

A negative ΔG implies a thermodynamically favorable complex formation, whereas a positive value implies an unfavorable one.

PyMOL graphical program was used to generate the molecular graphics images [7].

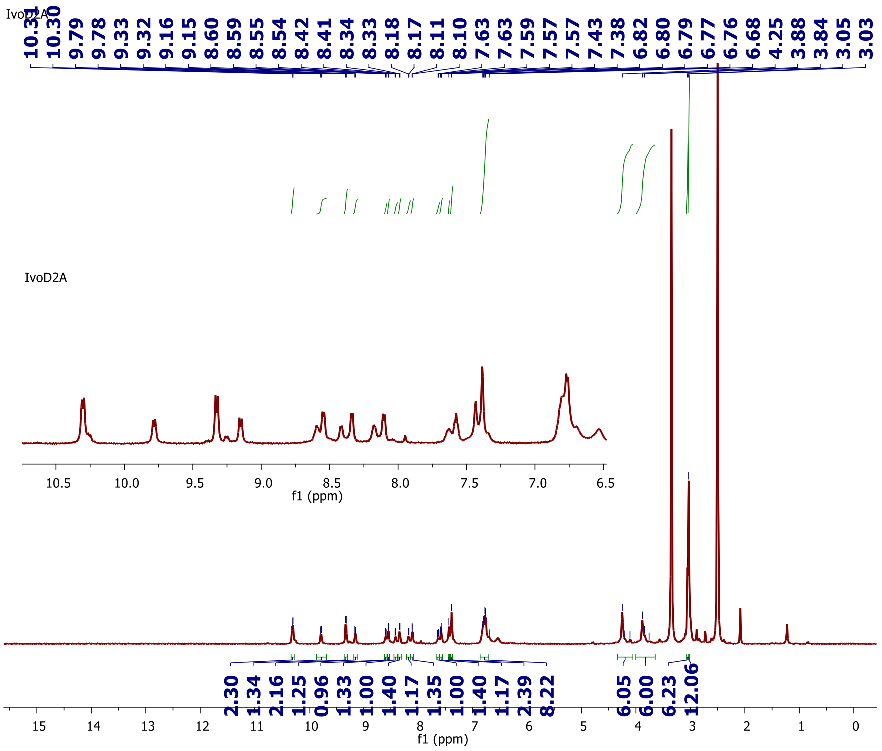

	Parameter	Value
1	Data File Name	E:/ after/ prof. Ivo/ results/ NMR/ IVO_NMR_21/ IvoD2A/ 11/ pdata/ 1/ 1r
2	Title	IvoD2A
3	Comment	
4	Origin	Bruker BioSpin GmbH
5	Owner	yavor
6	Site	
7	Spectrometer	spect
8	Author	
9	Solvent	DMSO
10	Temperature	293.0
11	Pulse Sequence	zg30
12	Number of Scans	32
13	Receiver Gain	287
14	Relaxation Delay	1.0000
15	Pulse Width	10.8000
16	Acquisition Time	1.7039
17	Acquisition Date	2021-11-24T18:40:45
18	Modification Date	2021-11-26T16:04:06
19	Spectrometer Frequency	599.98
20	Spectral Width	9615.4
21	Lowest Frequency	-252.0
22	Nucleus	1H
23	Acquired Size	16384
24	Spectral Size	65536

Figure S1. ^{1}H NMR of NI-DAT.

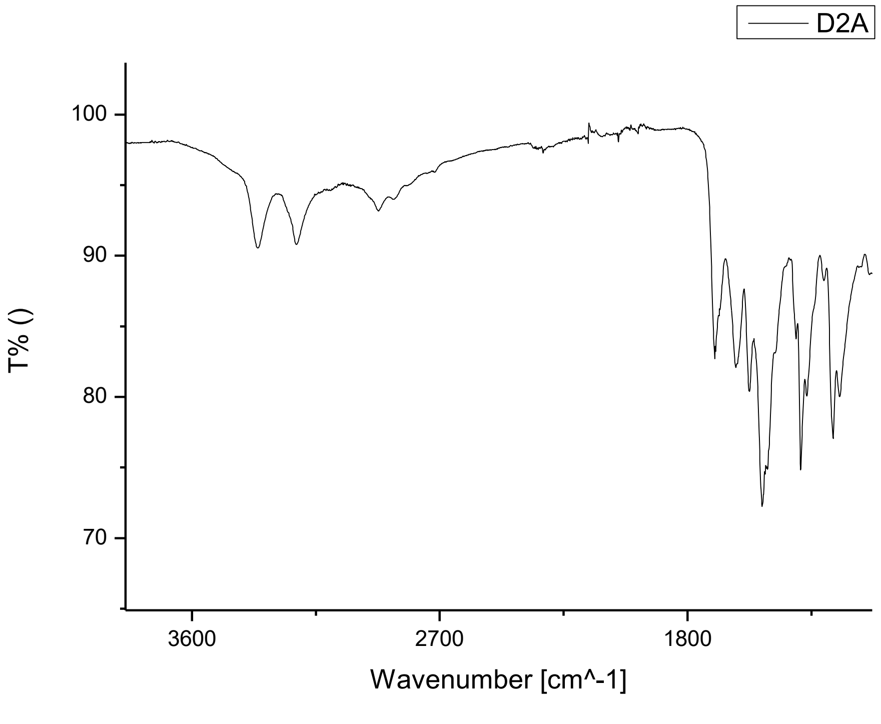

Figure S2. FTIR spectrum of NI-DAT.

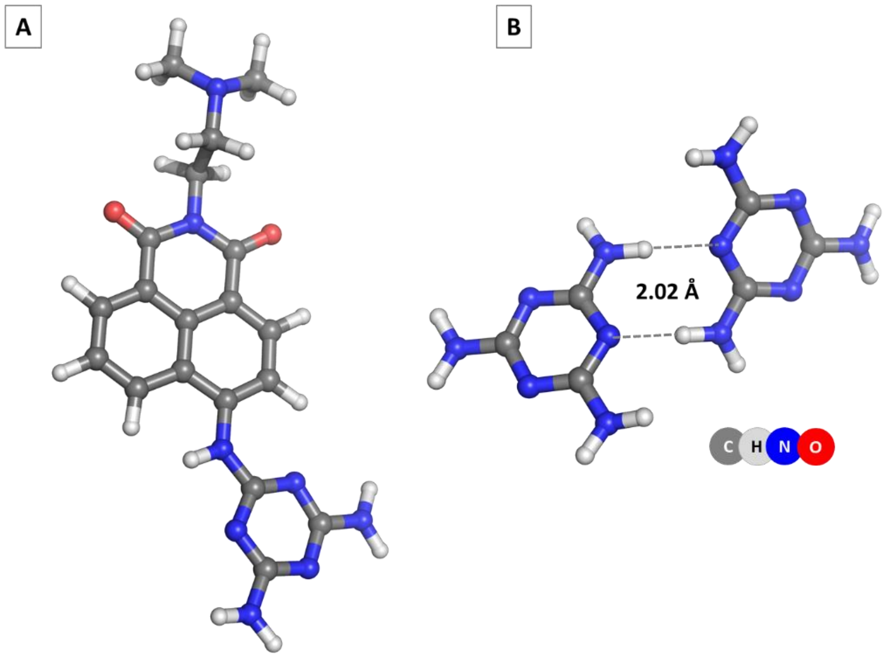

Figure S3. B3LYP/6-31+G(d,p) optimized structure of (**A**) NI-DAT and (**B**) dimer of TAT.

References

1. Frisch, M.J.; Trucks, G.W.; Schlegel, H.B.; Scuseria, G.E.; Robb, M.A.; Cheeseman, J.R.; Scalmani, G.; Barone, V.; Mennucci, B.; Petersson, G.A.; et al. *Gaussian 09*; Gaussian, Inc.: Wallingford, CT, USA, 2013.
2. Becke, A.D. Density-functional Thermochemistry. III. The Role of Exact Exchange. *J. Chem. Phys.* **1993**, *98*, 5648–5652, doi:10.1063/1.464913.
3. Lee, C.; Yang, W.; Parr, R.G. Development of the Colle-Salvetti Correlation-Energy Formula into a Functional of the Electron Density. *Phys. Rev. B* **1988**, *37*, 785–789, doi:10.1103/PhysRevB.37.785.
4. Hehre, W.J.; Lathan, W.A. Self-Consistent Molecular Orbital Methods. XIV. An Extended Gaussian-Type Basis for Molecular Orbital Studies of Organic Molecules. Inclusion of Second Row Elements. *J. Chem. Phys.* **1972**, *56*, 5255–5257, doi:10.1063/1.1677028.
5. Clark, T.; Chandrasekhar, J.; Spitznagel, G.W.; Schleyer, P.V.R. Efficient Diffuse Function-Augmented Basis Sets for Anion Calculations. III. The 3-21+G Basis Set for First-Row Elements, Li–F. *J. Comput. Chem.* **1983**, *4*, 294–301, doi:https://doi.org/10.1002/jcc.540040303.
6. Cancès, E.; Mennucci, B.; Tomasi, J. A New Integral Equation Formalism for the Polarizable Continuum Model: Theoretical Background and Applications to Isotropic and Anisotropic Dielectrics. *J. Chem. Phys.* **1997**, *107*, 3032–3041, doi:10.1063/1.474659.
7. Schrödinger, L.; DeLano, W. PyMOL, 2020. Available online: http://www.pymol.org/pymol (accessed on 5 June 2022).
